# Complex Craniofacial Cases through Augmented Reality Guidance in Surgical Oncology: A Technical Report

**DOI:** 10.3390/diagnostics14111108

**Published:** 2024-05-27

**Authors:** Alessandro Tel, Luca Raccampo, Shankeeth Vinayahalingam, Stefania Troise, Vincenzo Abbate, Giovanni Dell’Aversana Orabona, Salvatore Sembronio, Massimo Robiony

**Affiliations:** 1 Clinic of Maxillofacial Surgery, Head-Neck and NeuroScience Department, University Hospital of Udine, p.le S. Maria della Misericordia 15, 33100 Udine, Italy; 2 Department of Oral and Maxillofacial Surgery, Radboud University Medical Center, 6525 GA Nijmegen, The Netherlands; 3 Neurosciences Reproductive and Odontostomatological Sciences Department, University of Naples “Federico II”, 80131 Naples, Italy

**Keywords:** augmented reality, virtual surgical planning, navigation, sinonasal undifferentiated carcinoma, craniofacial resection, oncology

## Abstract

Augmented reality (AR) is a promising technology to enhance image guided surgery and represents the perfect bridge to combine precise virtual planning with computer-aided execution of surgical maneuvers in the operating room. In craniofacial surgical oncology, AR brings to the surgeon’s sight a digital, three-dimensional representation of the anatomy and helps to identify tumor boundaries and optimal surgical paths. Intraoperatively, real-time AR guidance provides surgeons with accurate spatial information, ensuring accurate tumor resection and preservation of critical structures. In this paper, the authors review current evidence of AR applications in craniofacial surgery, focusing on real surgical applications, and compare existing literature with their experience during an AR and navigation guided craniofacial resection, to subsequently analyze which technological trajectories will represent the future of AR and define new perspectives of application for this revolutionizing technology.

## 1. Introduction

Transposition of virtual digital plans into real patients during surgery has always represented a major problem, especially in cases of complex oncology procedures. Various technologies have been tested to solve this issue and translated into clinical practice, of which navigation is the most successful and widespread example, although it fails to provide a three-dimensional guidance and involves an expensive and cumbersome machine which requires the surgeon to modify their sight of the surgical field [1].

Augmented reality (AR) involves the projection of a computer-generated image into the real eyesight of the surgeon. In particular, optical see-through devices, including Hololens (Microsoft, Redmond, WA, USA) are the key to providing a wearable, comfortable device that can be used during surgery as a guidance to enhance anatomical understanding thanks to the simultaneous and superimposed vision of the virtual surgical planning [2]. Although this technology still lacks optimal calibration and therefore no solution is still certified for its use, it has been tested in a number of circumstances in head and neck surgery, including, orthognathic surgery, trauma, temporomandibular joint (TMJ), endoscopy and orbit [3,4]. However, no single use has been reported in complex oncologic surgery of the head and neck region, owing to its perceived limits and the complexity of optimizing a virtual surgical planning for such cases. Sinonasal undifferentiated carcinomas (SNUC) are rare high-grade epithelial neoplasms affecting the sinonasal cavity. Since its first description in 1986, only a few hundred cases of SNUC have been documented in medical literature [5]. The lack of known histogenesis and glandular or squamous differentiation caused it to be classified as a separate entity by the World Health Organization (WHO) [6]. It is more common in men, with a male to female incidence ratio of 2:1, and the age of presentation is typically in the fifth decade of life. [7] SNUC’s possible differential diagnoses include squamous cell carcinoma, olfactory neuroblastoma, neuroendocrine carcinoma, lymphoma, melanoma, rhabdomyosarcoma, and lymphoma [8]. Imaging techniques are not definitive in distinguishing SNUC from these conditions. Hence, the diagnosis of SNUC relies on examining tissue samples histopathologically, often involving immunohistochemical analysis. It is a highly aggressive neoplasm, with patients presenting to clinical attention at a very advanced stage, where fundamental structures of the skull base, brain, and orbit are already involved [9]. The initial symptoms of SNUC can seem harmless, like nasal blockage, nosebleeds, headaches, and facial discomfort. However, they may also manifest as vision problems, swelling around the eyes, proptosis, and cranial nerve palsies [9]. Cervical lymph nodes are involved in 10–30% of patients at the time of clinical presentation. The prognosis is bleak, also in view of the high rate of locoregional recurrences and distant metastases [10]. The treatment of choice is currently multimodal, with combinations of chemotherapy, radiotherapy and surgical resection. This latter remains the mainstay of treatment for SNUC [11]. However, due to the tumor’s proximity to critical structures like the orbit and skull base, performing surgery and ensuring complete removal of the tumor can be challenging. We conduct a narrative review about surgical applications of AR in maxillofacial surgery and we compare results of our experience to currently reported standards for craniofacial surgical oncology. The aim of this review is to widen perspectives for the clinical application of AR in a complex oncologic scenario represented by craniofacial resection of SNUC. Advantages of AR in this context are then discussed, emphasizing inherent limitations as well, and perspectives of possible future optimizations are presented to understand how this technology will become a valuable ally for complex head and neck surgical oncology.

## 2. Materials and Methods

This is a technical report presenting results of a case series enrolling 4 patients who underwent craniofacial oncologic surgery from January 2023 to November 2023. Patients’ demographic and clinical characteristics are described in Table 1. This study complies with ethical guidelines reported in the Declaration of Helsinki and was granted the approval number IRB_45_2020 by the Institutional Review Board of the University of Udine.

### 2.1. From Imaging to VSP

The first step for the digitalization of anatomy consists in performing adequate imaging techniques which can address both diagnostic and virtual surgical planning (VSP) needs [12]. For this reason, multiparametric imaging including both high-resolution CT (HRCT) and magnetic resonance (MR) sequences was acquired with well-defined spatial resolution criteria, including a voxel matrix of 512 × 512 px and a slice thickness of 1 mm for MR and 0.625 mm for HRCT. Several anatomical regions rely on a specific acquisition modality, including HRCT for reliable bone reconstruction with minimization of partial volume effect; time of flight (TOF) sequences to capture arterial blood flow through head and neck vasculature using flow void effect; volumetric interpolated breath-hold examination (VIBE) enables the creation of T1-weighted three-dimensional images within a 30 s breath-hold, allowing representation in detail of soft tissue anatomy in a short time interval and with spatial features much superior to traditional non-volumetric MR: this sequence is ideal for defining tumor boundaries, muscles, and moving fluids.

Once imaging is acquired in accordance with this protocol, multiple sequences must be coregistered within a unique coordinate system both using transformational algorithms that apply normalization and difference calculations between paired images and using a reference image. This ensures that all sequences match the same anatomical localization and is a crucial step to ensure the creation of a reliable presurgical model [13].

After coregistration, anatomy is digitized in 3D models (geometrical meshes) that are reconstructed through segmentation techniques, which involve a combination of threshold-based, free hand and semi-automatic brush techniques to include specific regions of the volumetric DICOM dataset in segmentation masks. Such masks are then converted into corresponding 3D triangulated representations, which can be entirely managed in an open software environment through universally accepted formats for computer graphics, including STereoLithography (STL) and Wavefront OBJect (OBJ).

Virtual surgical planning is entirely managed within the Materialise Mimics Innovation Suite version. 23.0 (Materialise, Leuven, BE), the most complete software package for surgical planning. Multiple osteotomies were traced according to the facial disassembly criteria for craniofacial resection, including a fronto-orbital access to the anterior skullbase and a transfacial approach using a rotational nasal-cheeck flap [14,15,16]. Each STL was assigned a different color pattern to be separately imported in the AR project.

### 2.2. Setting Up AR Project for VSP

Microsoft’s Mixed Reality Toolkit (MRTK) (Microsoft Corporation, Redmond, WA, USA) provides a key role to bridge virtual surgical planning with Hololens 2 as a device which supports augmented reality. In fact, MRTK also provides support for importing and working with custom 3D models, which are provided by VSP. Alternatively, Unity platform (Unity Technologies, San Francisco, CA, USA) can be used as an editor to build and then deploy the AR application based on VSP.

Interactable components are a core part of both Unity and MRTK and provide the basics to define a physical interaction between a virtual object and the physical space. It supports different types of input, including hand interactions and gestures as well as eye motion tracking.

Spatial Map Interaction represents another key feature of both Unity and MTRK and is based on a raycast from the camera along the camera’s viewing angle, providing a tool of interaction with the virtual surgical plan in the context of the real-world environment. There are additional technologies deserving to be mentioned which are implemented in such software models:

- Hand tracking: MRTK supports HoloLens 2 articulated hand tracking, allowing for direct hand interactions with virtual objects in the AR space. This can be used to manipulate the virtual surgical plan in a more intuitive and natural way [15].
- Eye tracking: Using built-in Hololens 2 sensors, MRTK also supports eye tracking. This can be used for more advanced interactions, enabling centering of the virtual surgical plan within the user’s sight field.
- Spatial anchors: Such tools enable fixing the AR model to any given reference of the physical space. Using Azure’s cloud technologies, such anchors can be moved to the cloud to adapt the same coordinate system between multiple devices.

Once the VSP is successfully transferred in the AR app and has been made interactable with both gaze and hand movements, it can be deployed and installed in the physical Hololens 2 headset.

### 2.3. Surgery

In these cases, given the need to perform a neck dissection, we proceed with a temporary tracheotomy, preferring a percutaneous tracheotomy to a surgical one. Patient preparation proceeds by positioning of the tracking system, usually in parieto-occipital region and the subsequential calibration of the navigator. Neck dissection can be performed before or after craniofacial resection of the malignant tumor. In the case of the patients under investigation, all underwent modified bilateral neck dissection. SNUC tends to mainly affect the maxillary sinus, so an en bloc resection is performed through various surgical accesses. After a Weber–Ferguson incision, a dissection plane is established and two osteo-myocutaneous flaps are prepared: a maxillary flap laterally and a nasogenial flap medially. Osteotomy of the bone portions affected by the tumour is then performed using a piezoelectric device, establishing the bony limits of the craniofacial resection. After pterygomaxillary disjunction, the tumor is excised. During these maneuvers, attention must be paid to possible severe bleeding, especially from the internal maxillary artery. When the neoplasm involves the frontal sinus, a coronal approach must be performed. A frontal bandeau above the superior orbital rim to allow access to the neoplasm and the anterior cranial fossa. Neuronavigation is crucial throughout surgery but particularly at the intracranial level, indicating and highlighting the limits of the neoplasm. An exenteratio orbitae is often required. Reconstruction of the defect often requires the use of local flaps, temporalis muscle flaps, or nasal septum flaps. Bony defects, e.g., in the frontal bone, are reconstructed using a titanium mesh. If it is necessary to restore the dural plane, a pericranial flap is normally used with a frontal sinus obliteration with fat. All patients then followed a protocol of adjuvant chemotherapy and radiotherapy.

## 3. Results

All patients were followed for the first year with monthly follow-up visits according to the oncology protocol of our clinic. No significant immediate postoperative complications were noted. All patients underwent adjuvant radio-chemotherapy. Patients underwent follow-up MRI between 2 and 4 months post-operatively. To date, there has been no evidence of recurrence.

## 4. Discussion

Augmented reality is advocated as a future game-changer in complex surgical scenarios. The availability of a virtual image overlapped to the real patient can provide a number of benefits, including continuous guidance, real-time scoping of the complex three-dimensional anatomy, lesion targeting, and avoidance of vulnerable structures [1,2]. Such possibilities will have a consistent impact on complex craniofacial oncology surgeries, and they will act as the bridge to transpose virtual surgery performed preoperatively in a software environment within the operating theatre.

Currently, AR is being increasingly applied in CMF surgery. Reported evidence in the literature is mostly limited to in vitro settings, where phantom models are used to test calibration methods or to compare novel AR interfaces with navigational stations in terms of accuracy [17,18,19]. Moreover, there is no standardized solution for AR, as wearable devices, smartphone or tablet app, and composite circuits made of connected cameras and laptops have been interchangeably reported [20].

Evidence of AR application in a real surgical setting is scant in the literature. This can be explained considering that AR adoption is recent and still largely unreliable for a validated clinical use owing to a number of reasons, primarily including the inherent imprecision in patient-to-VR (virtual reality) matching, parallax error, gross environmental mapping, light interference, patient position, and sterile drapes concealing the anatomical region used for calibration. Nevertheless, there is some speculative research trying to explore the possible advantages of AR in surgical scenarios as well as hardware ergonomics in terms of wearability, interference with surgical maneuvers, vision through optical lenses with overlapped display for VR, mesh rendering, and overall fluidity.

Table 2 collects the few reports presenting examples of AR in a real surgical setting. Despite the obvious lack of a regulatory framework for this early technology, some applications have been tested using a combination of commercially available or custom-built hardware and a variety of software interfaces.

Among the very few reports dealing with augmented reality, there is not a single paper trying to implement this promising technology in complex oncologic craniofacial procedures. Although in such an initial stage the implementation of AR in this setting is mainly conceptual, its practical adoption may provide valuable insights in the perception of the value that this technology may have in the future and how it may be supposed to enter the clinical routine.

Our group started to speculate on AR for complex craniofacial procedures, among which open craniofacial resection for advanced-stage sinonasal tumors represented the ideal setting of application. Virtual surgical planning was described according to the protocol of digital anatomical reconstruction for deep facial compartments described by the same authors [24]. A multilayer segmentation method was applied for the separate reconstruction of bone, segmented using a thresholding algorithm on CT scans, whereas, for soft structures, multiple MR sequences performed with a 3T magnet were coregistered and matched with the CT and allowed to reconstruct the tumor mass and critical vascular structures, both arterial, using time-of-flight volumetric and venous sequences using a 3D venography sequence to track the centripetal proton flow (Table 3).

**Figure 1:**
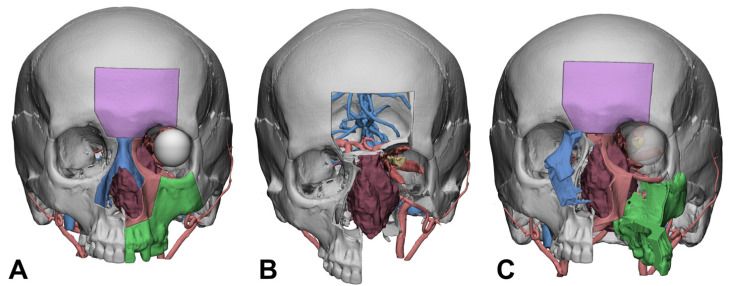
Virtual surgical planning for facial disassembly to be imported in AR headset. (**A**) osteotomies are traced in VSP software; (**B**) all bone flaps are removed (purple—frontal bone flap; blue—nasal bridge; red—subspinal medial orbital wall flap; green—maxilla flap), leaving the tumor (brown) visible in its relationships with the surrounding structures; (**C**) facial translocation is simulated, pivoting and rotating bone segments according to the surgical prediction.

Based on the segmented virtual anatomy, a virtual patient replica was generated and could be further processed for topological optimization of 3D models. Virtual surgical planning of osteotomies was conducted by CMF specialists according to the procedure described by Ketcham [27], including the simulation of an orbito-frontal bandeau to access the anterior cranial fossa and nasal bone and maxillary osteotomies for the midfacial translocation approach described by Janecka and Tiedemann [28]. This allowed us to split the midface in a maxillary cheek flap and a contralateral nasal–maxillary cheek flap (Figure 1).

**Figure 2:**
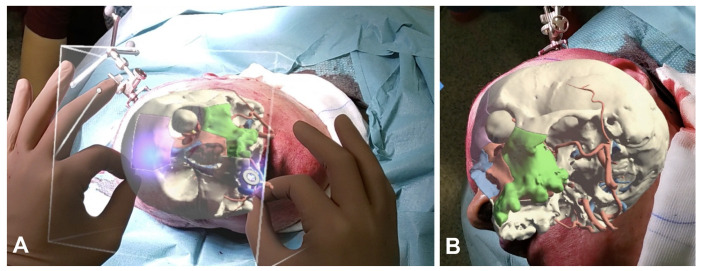
(**A**) interactive overlap of the AR image with the surgical field; (**B**) observing the alignment from multiple perspectives while the AR projection remains stable in space. However, a slight parallax error is present; You can see the individual objects divided and colored as shown in table one.

The models with facial disassembly according to planned osteotomies were exported either to create a 3D printed replica, and to be imported in the navigation plan. We used for this purpose a novel commercially available platform, Mimics Viewer XR (Materialise, Leuven, BE, USA) which allowed us to create an AR plan suitable for Hololens 2 glasses (Microsoft, Redmond, WA, USA). The viewer provides a real time rendering engine without any graphic detail loss and is well integrated with Hololens hand tracking for fine motion gestures that enable the user to manipulate the virtual object in real space. Moreover, with a simple gesture, the user can display a pop-up menu allowing them to selectively show and hide parts, in order to dismantle the craniofacial skeleton according to the virtual surgical plan, as well as cropping the model to scope in-depth anatomy, including skull base and deep facial compartments. As this is a preliminary investigation based on a single experience, AR was not used as a real guidance to perform osteotomies, which were instead traced under navigational control. At first, Hololens software performs an environmental recognition to determine the conformation of surrounding space, including floor and wall recognition. This is achieved using embedded coupled infrared cameras that are natively inserted in the headset and ensures that the virtual object is located stably in the physical environment. Then, the AR model is manually positioned over the real patient, using anatomical prominences as reference. A soft tissue model provides the best alignment template as it matches the size and shape of the patient. The model is overlapped using fine gestures to the real patient from multiple sight angles to compensate for the parallax error and, once the surgeon is satisfied with the registration, the soft tissue mask is hidden, leaving the skeletal surgical plan and the tumor visible, as well as the area marked for resection (Figure 2).

This system is at its earliest development phase but, compared with traditional navigation systems, it provides unprecedented ergonomics and simplicity, as the surgeon does not need a navigation pointer or a cumbersome infrared camera in the operating theatre. Moreover, wearing the AR headset makes it unnecessary to modify head position or to shift gaze from the operating field to look at the navigation monitor [29].

There are of course multiple drawbacks that still must be approached from an engineering perspective—not just parallax error but also eye movements and discrepancy between a focal plane and an object can render the image misaligned. Current-generation AR devices implement a single focal plane, which increases such discrepancy between the observer’s focal plane and the object’s distance. Newer devices will integrate eye-tracking technology by measuring eye positions and eye movement to account for the user’s viewing positions. In addition, the AR image perceived on Hololens is flat and lacks depth of field, and this is likewise due to the fact that a single focal plane restricts the user to focus the virtual image exclusively at a fixed distance, leading to eye fatigue. Depth perception is essential to determine the penetration of surgical instruments within the overlaid surgical planning in a real operative field, and the addition of multiple focal planes might significantly enhance the performance of AR headsets [30].

However, especially in complex craniofacial surgery, the operative field changes continuously due to different phases of surgical operations, head movements, light conditions, and instrumentation. Therefore, it is foreseeable that in the future an interactive method of AR-to-patient registration will become necessary, which will be able to readapt calibration of the virtual image on the variations of the operative field. For this purpose, deep learning and convolutional neural networks are the most promising approaches, as such algorithms will “learn” from the different surgical phases, will elaborate a novel calibration, and will provide the new output back to the headset, thus enabling a real-time virtual image processing [31]. Similarly, subject recognition capabilities will substantially improve using deep learning, including the possibility to adapt recognition to the progression of surgical steps and the modification of anatomy, as a midfacial split overturns completely any landmark registered before.

Moreover, novel AR system will provide the user with visual feedback options, as suggested by Maal et al. [31], showing the user an immediate clue on the correctness of surgical maneuvers, such as lesion targeting or osteotomies [32].

Finally, AR applied in craniofacial surgical oncology, but also in endoscopic approaches [33], is likely to become a fundamental resource to enhance anatomy understanding and lesion identification and to avoid critical structures using a see-through, wearable display. This preliminary report aimed to present the importance of progressively introducing AR to enhance surgical orientation in complex scenarios, a process that will be fostered as soon as certification authorities validate AR systems for clinical use.

The main limitation of this study is that it collects AR experience at its earliest, given the fact that few solutions are available in the market for certified use. The main problem still consists in the correct calibration of the holographic image with the reality of the patient and the physical environment, although artificial intelligence will definitely help to overcome this limitation.

## 5. Conclusions

The importance of AR is growing in modern craniofacial surgery and is steadily shifting from mere research interest to practical application. Especially in cases of complex surgical oncology procedures, the guidance provided by AR may be helpful in resolving scenarios in which the tumor mass is located in deep spaces or in proximity of crucial structures. We urge technological innovation and especially artificial intelligence to enhance the reliability of AR and translate it into a standard of care for the new generations of surgeons.

## Figures and Tables

**Table 1:** Patients’ demographic and clinical data.

Patient ID	Gender	Age	Pathology
1	Male	56	SNUC involving the right maxillary sinus, extending to the nasal cavity right ethmoid sinus and retro maxillary space
2	Female	69	SNUC involving the left maxillary sinus eroding the medial, lateral and superior wall breaching through the orbit.
3	Male	62	SNUC involving the nasal cavity with extension to the ethmoidal and frontal sinus, eroding the right medial orbital wall
4	Female	73	SNUC involving the right maxillary sinus extending to the right nasal cavity into ethmoid, sphenoid, and frontal sinus and through cribriform plate.

**Table 2:** Review of studies presenting clinical applications of augmented reality in real surgical operations, documented by pictures.

Author and Year	Surgery	Hardware	Software
Ahn et al., 2019 [21]	Orthognathic	Coupled cameras, processing station, markers	Custom built software in C++
Pham Dang et al., 2021 [22]	Maxillary cyst excision	Single DSLR camera	open-source libraries Qt, OpenCV and VTK (Visualization Tool Kit)
Battaglia et al., 2019 [3]	Fibula flap harvesting	Smartphone-Tablet	Unity 3D
Lysenko et al., 2022 [23]	Maxillary cyst excision	Microsoft Hololens	Not declared
Ceccariglia et al., 2022 [2]	Oncologic surgery	Microsoft Hololens	Unity 3D + VuForia engine; UWP app

**Table 3:** Multilayer anatomical segmentation and techniques for each structure [25,26].

Anatomical Structure	Imaging Acquisition Modality	Segmentation Method	Software Used for Segmentation	Software Used for Postprocessing
Skull and mandible	hi-res CT scan	Gobal thresholding and mask split	Materialise Mimics	Materialise 3-matic
Eye	T2 MRI	Local region growing and voxel dilation	Materialise Mimics	Materialise 3-matic
Arterial system	TOF (3T)	Dynamic region growing + vessel tracking	Materialise Mimics	Materialise 3-matic
Venous system	3D venography (3T)	Dynamic region growing	Materialise Mimics	Materialise 3-matic
Tumor	VIBE volumetric	Smart brush + manual	Materialise Mimics	Materialise 3-matic

## Data Availability

The data presented in this study are available on request from the corresponding author. The data are not publicly available due to privacy reasons.
